# Sleep Facilitates Problem Solving With No Additional Gain Through Targeted Memory Reactivation

**DOI:** 10.3389/fnbeh.2021.645110

**Published:** 2021-03-03

**Authors:** Felipe Beijamini, Anthony Valentin, Roland Jäger, Jan Born, Susanne Diekelmann

**Affiliations:** ^1^Federal University of Fronteira Sul, Realeza, Brazil; ^2^Institute of Medical Psychology and Behavioural Neurobiology, University of Tübingen, Tübingen, Germany; ^3^Werner Reichardt Centre for Integrative Neuroscience, University of Tübingen, Tübingen, Germany; ^4^Department of Psychiatry and Psychotherapy, University Hospital Tübingen, Tübingen, Germany

**Keywords:** sleep, slow wave sleep, targeted memory reactivation, problem solving, memory

## Abstract

According to the active systems consolidation theory, memories undergo reactivation during sleep that can give rise to qualitative changes of the representations. These changes may generate new knowledge such as gaining insight into solutions for problem solving. targeted memory reactivation (TMR) uses learning-associated cues, such as sounds or odors, which have been shown to improve memory consolidation when re-applied during sleep. Here we tested whether TMR during slow wave sleep (SWS) and/or rapid eye movement (REM) sleep increases problem solving. Young healthy volunteers participated in one of two experiments. Experiment 1 tested the effect of natural sleep on problem solving. Subjects were trained in a video game-based problem solving task until being presented with a non-solved challenge. Followed by a ~10-h incubation interval filled with nocturnal sleep or daytime wakefulness, subjects were tested on the problem solving challenge again. Experiment 2 tested the effect of TMR on problem solving, with subjects receiving auditory TMR either during SWS (SWSstim), REM sleep (REMstim), or wakefulness (Wakestim). In Experiment 1, sleep improved problem solving, with 62% of subjects from the Sleep group solving the problem compared to 24% of the Wake group. Subjects with higher amounts of SWS in the Sleep group had a higher chance to solve the problem. In Experiment 2, TMR did not change the sleep effect on problem solving: 56 and 58% of subjects from the SWSstim and REMstim groups solved the problem compared to 57% from the Wakestim group. These findings indicate that sleep, and particularly SWS, facilitates problem solving, whereas this effect is not further increased by TMR.

## Introduction

Sleep is essential for adequate cognitive functioning and particularly for the consolidation of newly acquired memories (Durmer and Dinges, 2005; Rasch and Born, 2013). According to the active systems consolidation theory, memory consolidation during sleep relies on the spontaneous repeated reactivation of neural representations from previous learning experience (Ji and Wilson, 2007; Diekelmann and Born, 2010). This memory reactivation during sleep is assumed to have a two-fold function, on the one hand stabilizing and strengthening initially labile memory to representations making them less susceptible interference (Ellenbogen et al., 2009), and on the other hand reorganizing and integrating new memories into the pre-existing network of long-term memories in a process of system consolidation (Inostroza and Born, 2013; Stickgold and Walker, 2013; Schonauer et al., 2014). System consolidation and the associated restructuring of memory networks during sleep can thereby lead to qualitative changes of memory representations, for example allowing for the development of cognitive schemas (Lewis and Durrant, 2011; Landmann et al., 2014), the abstraction of gist knowledge (Payne et al., 2009; Diekelmann et al., 2010; Lutz et al., 2017) and the extraction of statistical regularities (Ellenbogen et al., 2007; Durrant et al., 2011).

By restructuring and changing memory representations, sleep may also aid the generation of insight and creative solutions for problem solving (Sio and Ormerod, 2009; Lewis et al., 2018), although evidence for this assumption is mixed. In one of the first studies on this question, Wagner and colleagues reported higher rates of insight into hidden rules in the Number Reduction Task (NRT) when participants slept after a first practice on the task compared to when they stayed awake during this post-practice period (Wagner et al., 2004). Monaghan and colleagues found increased analogical transfer after sleep, with subjects showing better abilities of applying previously learned problem solutions to structurally similar new problems (Monaghan et al., 2015). In the Remote Associates Test (RAT), in which subjects are asked for a common semantic association of three presented words, sleep likewise increased the chance of finding the solution (Cai et al., 2009), although this effect may only be evident for difficult but not easy RAT problems (Sio et al., 2013). However, other studies found no effects of sleep on a number of different problems, including change detection, riddles, anagrams, magic tricks and classical problem solving tasks like the Nine-dot problem (Brodt et al., 2018; Schonauer et al., 2018). The role of single sleep stages for problem solving is also unclear, with some findings suggesting beneficial effects of slow wave sleep (SWS) (Yordanova et al., 2008, 2012), while others observed associations with rapid eye movement (REM) sleep (Cai et al., 2009). This mixed evidence points toward differential effects of sleep and single sleep stages for problem solving, presumably depending on the type of problem task and associated underlying processes of solution generation (Lewis et al., 2018; Lerner and Gluck, 2019; Lutz and Born, 2019).

In problem solving, two distinct types of problems can be distinguished, with divergent problems requiring many different and sometimes unusual and creative answers, whereas convergent problems are characterized by only one correct solution (Guilford, 1967). For convergent problems, the correct solution can be reached either in an analytical way, with a conscious strategical step-by-step approach to reach the solution, or as a sudden understanding how to solve the problem, relying on unconscious processing that culminates in sudden insight (Bowden et al., 2005; Kounios and Beeman, 2014; Salvi et al., 2016; Laukkonen and Tangen, 2018). Insight problem solving thereby relies on the ability to relax certain task constraints and to form novel connections between existing concepts (Knoblich et al., 1999; Ormerod et al., 2002; Ollinger et al., 2013). One of the tasks that are characterized to a large extent by complex insight problem solving, is the ‘Beijamini version of the Speedy Eggbert Mania® video game' (B-SEM task) that has been previously applied by Beijamini et al. (2014). In this task, subjects command a video game character in different scenarios to interact with certain tools, like a crane, to move boxes around in order to reach a goal. In one of the scenarios, at some point, the goal can no longer be reached by moving boxes, but the crane has to be used to pick up and move the character itself across an obstacle. Arriving at this solution is often described by subjects as a sudden insight accompanied by an *Aha!* experience (Kaplan and Simon, 1990; Bowden et al., 2005; Topolinski and Reber, 2010). Applying this task, Beijamini et al. (2014) found that sleep doubled the chance to find the solution compared to a period of wakefulness. Furthermore, subjects who obtained SWS during the sleep period had a higher chance of solving the problem, implicating SWS in the generation of problem solutions. However, that previous study also had some limitations. First, it only evaluated the effect of a short nap of about one hour, leaving open the question whether a full night of sleep yields similar or even larger benefits. Secondly, because of the short sleep duration and an overall low amount of SWS, the association of problem solving with the amount of SWS could not be properly tested. Thirdly, pre-sleep performance levels varied strongly across subjects because each subject was allowed to play as many levels of the video game task as possible until being unable to solve it, making comparisons between subjects difficult. In the first part of the present study, we aimed at replicating and extending the findings by Beijamini et al. (2014). In Experiment 1, we tested the effects of a whole night of sleep with larger amounts of SWS on problem solving in a more standardized version of the B-SEM task, with all subjects playing the same levels resulting in similar pre-sleep performance. We expected higher problem solution rates after a whole night of sleep compared to wakefulness, and we hypothesized problem solving to be associated with the amount of SWS.

In the second part of the present study, we asked whether targeted memory reactivation (TMR) during sleep facilitates problem solving on the B-SEM task. TMR is a technique that has been frequently applied to manipulate memory reactivation processes during sleep by pairing learning experiences with specific cue stimuli (like sounds or odors) and re-applying those same cue stimuli again during subsequent sleep. TMR has been shown to bias reactivation processes during sleep toward the cued contents (Bendor and Wilson, 2012) thereby facilitating memory consolidation in a wide range of tasks (Klinzing and Diekelmann, 2019; Hu et al., 2020) as well as generalization and extraction of explicit knowledge from implicitly learned materials (Cousins et al., 2014; Diekelmann et al., 2016; Batterink and Paller, 2017). However, little is known about whether TMR can also boost problem solving during sleep. In two recent studies, Ritter and colleagues found more creative solutions in a divergent problem solving task after TMR with problem-associated odors during sleep (Ritter et al., 2012), and Sanders and colleagues observed higher solution rates for previously unsolved puzzles after TMR with puzzle-associated sounds during sleep, specifically during SWS (Sanders et al., 2019). Yet, it remains unclear whether TMR benefits problem solving in the more complex convergent “insight-like” B-SEM task and whether this effect depends on a specific sleep stage. Therefore, in Experiment 2, we compared auditory TMR with video game-related sounds presented during SWS or during REM sleep with a wake control condition. Based on previous evidence (Beijamini et al., 2014; Sanders et al., 2019), we expected higher problem solution rates after TMR during SWS then during REM sleep or wakefulness.

## Methods and Materials

### Participants

All participants were healthy non-smokers between 18 and 30 years of age, had no history of endocrine, sleep, neurological, or psychiatric disorders, no history of drug or alcohol abuse, did not take any medication at the time of the experiment (except for oral contraception pill) and abstained from night shift work for at least 6 weeks prior to the experiment. For the 24 h before and the time during the experiment, subjects were instructed to refrain from alcohol, caffeine, drugs, napping, and taking medication (other than oral contraception pill), which was monitored *via* questionnaire. In the night before the experiment, participants were instructed to sleep no <7 h and no > 9 h. Subjects were randomly assigned to the experimental groups (in Experiment 1: Sleep and Wake group; in Experiment 2: SWSstim, REMstim, and Wakestim groups). All subjects were paid for participation and gave written informed consent. The study was approved by the local ethics committee of the medical faculty of the University of Tübingen (IRB 623/2014BO2).

Participant recruitment was done *via* the university mailing list at the University of Tübingen. Volunteers received detailed information about the study as well as a screening questionnaire *via* email and were asked to fill out the questionnaire and return it to the experimenter. Depending on the volunteers' answers with regard to inclusion and exclusion criteria, they were then informed about whether or not they would be suitable for participation. Overall 56 volunteers participated in Experiment 1. Fourteen participants were excluded from the final analyses because of solving the challenge already during the practice session (4), reporting having had the idea for the solution before falling asleep (2), practicing the task during the incubation interval (1), taking medication on the experimental day (2), resigning from the experiment (3), having difficulties with sleeping (1), and technical issues with EEG recording (1). For the remaining 42 subjects (Sleep group: *n* = 21, Wake group: *n* = 21), there were no differences between groups in sex distribution (χ^2^ = 0.62 *p* = 0.43), age [*t*_(40)_ = −1.00; *p* = 0.32] or usual amount of sleep [*t*_(__40)_ = −0.44; *p* = 0.64; see Table 1]. Overall 81 volunteers participated in Experiment 2. 23 participants were excluded from the final analyses because of solving the challenge during the practice session (10), reporting having had the idea for the solution before falling asleep (1) or right after the practice session (1), practicing the task during the incubation interval (1), resigning from the experiment (2), having difficulties sleeping (1), technical issues with TMR (3) or the video game task (3) or the four-choice reaction time task (1). For the remaining 58 subjects (SWSstim group: *n* = 18, REMstim group: *n* = 19, Wakestim group: *n* = 21), there were no differences between groups in sex distribution (χ^2^ = 0.30; *p* = 0.86), age [*F*_(2, 55)_ = 0.17; *p* = 0.85] and the habitual sleep duration [*F*_(2, 54)_ = 0.94; *p* = 0.40; Table 1].

**Table 1:** Description of the sample for Experiment 1 and Experiment 2.

	**Experiment 1**		**Experiment 2**		
	**Wake**	**Sleep**	**Wakestim**	**SWSstim**	**REMstim**
N (female)	21 (18)	21 (16)	21 (17)	18 (14)	19 (14)
Age	24.3 ± 2.8	23.3 ± 3.9	22.9 ± 2.5	22.5 ± 2.0	22.8 ± 2.7
Usual sleep	7.7 ± 0.4	7.7 ± 0.5	7.8 ± 0.5	8.0 ± 0.5	8.0 ± 0.5

*Values for age and usual amount of sleep (in hours) are presented as means ± standard deviation*.

### Design and Procedure

In both experiments, participants from the sleep groups spent an adaptation night in the laboratory prior to the experimental night, including placement of the electrodes for polysomnographic recordings and sleep scoring to ensure absence of abnormal sleep and/or EEG features. On the day of the experiment, all participants took part in a practice session, an incubation interval (filled with sleep or wakefulness) and a test session.

**Figure 1:**
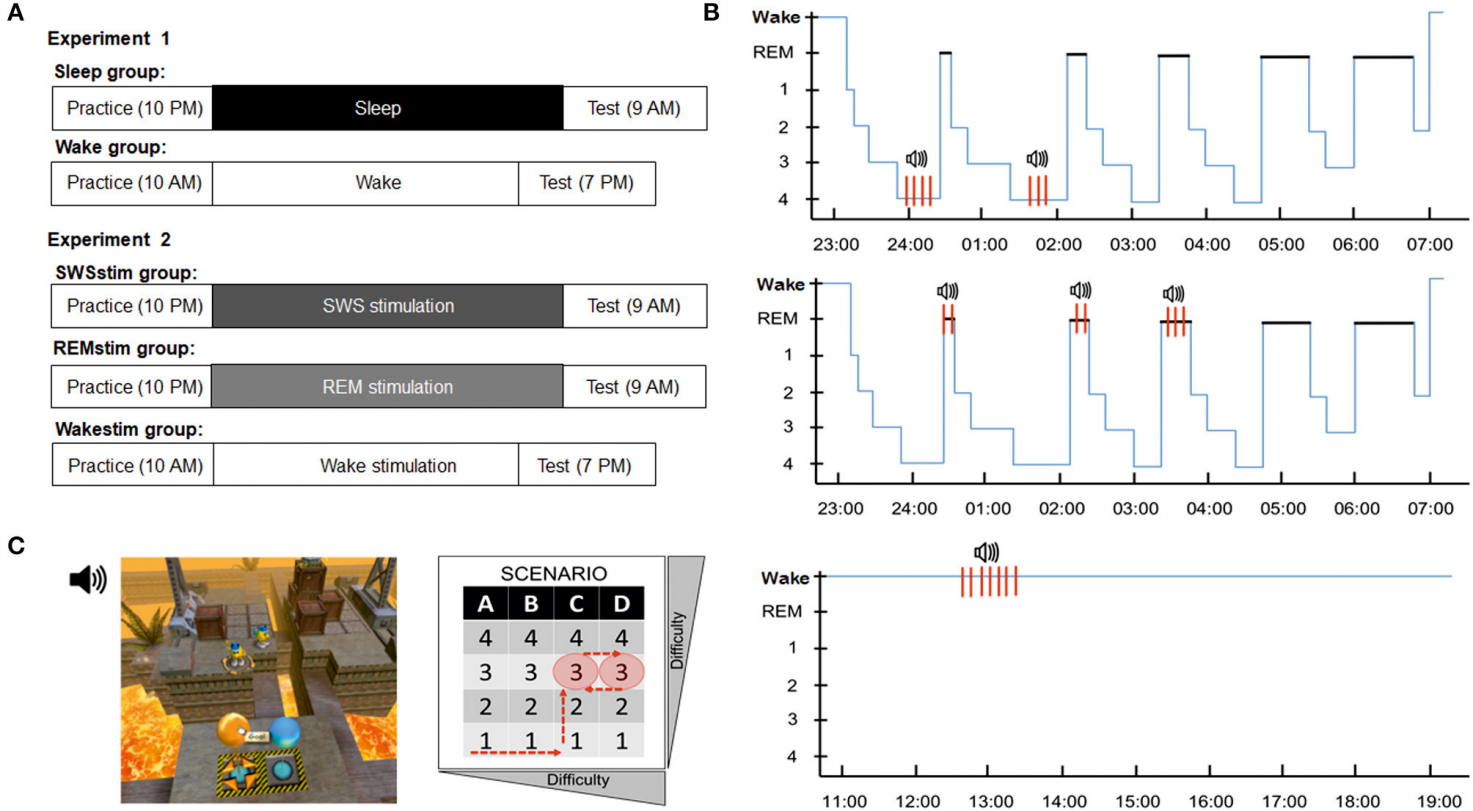
Experimental design and procedures. **(A)** In Experiment 1, subjects in the Sleep group practiced the problem-solving task in the evening, spent a whole night of sleep in the lab and were tested on the task again in the morning. The Wake group practiced the problem-solving task in the morning, spent the day awake and was tested in the evening. In Experiment 2, subjects in the SWSstim group and the REMstim group practiced the task in the evening, slept a whole night in the lab with auditory targeted memory reactivation (stimulation) either during SWS or REM sleep and were tested in the next morning. Subjects in the Wakestim group practiced the task in the morning, spent the day awake while they received targeted memory reactivation, and were tested in the evening. **(B)** Targeted memory reactivation protocols in the SWSstim group (top), the REMstim group (middle) and the Wakestim group (bottom). **(C)** Visual depiction of the B-SEM task layout (left). The player controls the movement and actions of a character by mouse to reach a floating balloon that will bring him to the next level. To reach this goal the player needs to command the character to interact with different objects and tools in limited 3D scenarios. The task includes four different scenarios with increasing task difficulty, each with four difficulty levels (right). All subjects played four levels during initial practice in fixed order: level 1 of scenario A, level 1 of scenario B, level 1 of scenario C, level 2 of scenario C. Finally, subjects played level 3 of scenario C, representing the problem solving level. None of the subjects in the final analysis solved this level during practice. At testing, all subjects played two levels: the unsolved level 3 of scenario C from the practice session and the new level 3 of scenario D, with the order of levels being counterbalanced across subjects.

In Experiment 1, two groups of participants were examined (Sleep group and Wake group; Figure 1A). In the Sleep group, subjects arrived at the sleep lab at ~20:30 and were prepared for polysomnography. Then, they filled out questionnaires and performed on the control tasks before the practice session of the video game (including the problem solving task) started at ~22:00. After the practice session, subjects filled out a game experience questionnaire and then went to bed at ~23:00 and were allowed to sleep undisturbed. Eight hours after lights off, subjects were awakened and electrodes were removed. The test session started at least 30 min after awakening to avoid sleep inertia. Subjects first completed another set of questionnaires and the control tasks before performing the video game (including the problem-solving task) again. Upon completion, subjects filled out the game experience questionnaire again and those subjects who did not solve the task during the test session were shown the solution. All subjects then left the lab. The Wake group followed essentially the same protocol. Subjects arrived at the lab at ~10:00, filled out the questionnaires, performed on the control tasks and then practiced the video game with the problem-solving task. After the practice session, they filled out the game experience questionnaire and were then allowed to leave the lab and go about their daily activities. They were instructed to avoid any strenuous activities, physical or mental (e.g., no exams, no extreme sports), and to refrain from napping. They returned to the lab at 19:00 for the test session, which was identical to the Sleep group.

In Experiment 2, three groups of participants were examined (SWSstim group, REMstim group, and Wakestim group; Figure 1A). In the SWSstim group and the REMstim group, subjects arrived at the sleep lab at ~20:30 and were prepared for polysomnography. For the practice session, they followed the same protocol as in Experiment 1, including questionnaires, control tasks and the video game problem solving task. After the practice session, subjects went to bed at ~23:00 with in-ear headphones taped to their ears. Sound calibration of white noise ensued, which was then continuously presented throughout the night at 36 dB. Subjects were informed that they might hear some sounds during the night. Following lights off, polysomnographic recordings were scored online by the experimenter and application of the (TMR protocol was applied during SWS for the SWSstim group and during REM sleep for the REMstim group, respectively (see below, Figure 1B). Subjects were awakened after 8 h of sleep and the test session started at least 30 min after awakening like in the Sleep group of Experiment 1.

The Wakestim group followed essentially the same protocol as the SWSstim and REMstim groups but arrived at the lab at ~10:00 for the practice session. Following the practice session, white noise of 36 dB was started and presented continuously, while participants watched the first 45 min of ‘The Lion King 2: Simba's Pride'. After 45 min, the movie was stopped and subjects were instructed to perform on a four choice reaction time task. In this task, subjects were asked to respond as fast and as correctly as possible to a crosshair appearing randomly in one of four squares arranged horizontally on the screen by pressing one of four corresponding buttons. The task consisted of 5 runs with 500 trails each and 30-s breaks between runs. Subjects were informed that the task would be accompanied by sounds played in the background *via* in-ear headphones. The TMR protocol was identical to the SWSstim and the REMstim groups. After completing the TMR protocol, subjects were allowed to leave the lab and go about their daily activities like in the Wake group of Experiment 1. They returned to the lab at 19:00 for the test session, which was identical to the other groups.

### Targeted Memory Reactivation

In Experiment 2, all groups received the same auditory targeted memory reactivation (TMR, sound stimulation) protocol. It consisted of a constant chain of 22 one-second sound tracks, starting and finishing with cue tones. The cue tones were bell/ring sounds, which were not encountered during any other part of the experiment. The other 20 sounds were audio snippets from a recording of the video game problem solving task (e.g., sounds of bubbling lava, steam sounds by the crane, running footsteps). The entire audio chain was repeated 10 times, such that the TMR stimulation protocol lasted overall ~22 min. In the SWSstim group and the REMstim group, the TMR protocol was played during the first 2–3 SWS and REM sleep periods, respectively. Limiting TMR to the first couple of sleep periods is a standard procedure to (1) maximize beneficial effects of TMR, given that neuronal replay and associated mechanisms such as slow wave activity in SWS and possibly also theta activity in REM sleep are most pronounced during the first sleep periods (Dijk and Czeisler, 1995; Bjorness et al., 2018), and (2) to minimize potential adverse effects of TMR such as inducing arousals and waking up upon sound presentation due to reduced sleep depth in later sleep periods. The TMR protocol was stopped whenever the sleep stage changed or arousals were detected. Stimulation resumed upon detection of stable SWS and REM sleep again, respectively. The sounds were presented at a volume of ~48–60 dB on top of the white noise played at 36 dB. In the Wakestim group, the sounds were presented during performance on the four choice reaction time task. White noise was played continuously at 36 dB and the sounds were presented on top of that at ~55–56 dB. Sound stimulation was presented only during the 5 runs of the four choice reaction time task and was paused during the 30-s breaks.

### Problem-Solving Task

The B-SEM task is an adaptation of the video game Speedy Eggbert Mania® and similar to the task applied by Beijamini et al. (2014). On this task, the player controls the movement and actions of an egg-shaped character named “Blupi” by mouse to reach a floating balloon that will bring him to the next level. To reach this goal the player needs to command Blupi to interact with an assemblage of boxes by moving them directly or indirectly throughout a limited 3D scenario. As the player progresses through the scenarios, the complexity and variety of available objects increases. The game features four different scenarios (A–D), each with four different levels (1–4) according to increasing difficulty (Figure 1C). Each scenario features specific ambient sounds that match the setting (e.g., bubbling of lava, birds chirping, thunder, rain, and running footsteps).

As an adaptation of the protocol from Beijamini et al. (2014), in the present study all subjects played the same levels of the video game, allowing for quantitative comparisons of performance. During the practice session, subjects were presented with the video game environment and played four different levels in order to learn how to play the game (Figure 1C). If certain check points were reached, the experimenter gave advice to the subjects, helping them to finish all four practice levels. After playing the four levels, subjects were presented with a different level (level 3 of scenario C), in which they were given 10 min of free trial to solve this challenging level (problem-solving task), without any help from the experimenter. The solution to the problem was of a sudden insight type, since subjects were required to apply previously learned strategies in a completely different way. Specifically, instead of the character moving boxes around with the help of different tools (e.g., cranes), the solution was to pick up and move the character itself by using a crane. Once subjects had figured this out, the rest of the solution was not different from the practice levels. Subjects who solved the problem (within the 10-min interval) were dropped from the study (this was the case for two subjects in the Sleep group, two subjects in the Wake group, four subjects in the WakeStim group, three subjects in the SWSstim group and three subjects in the REMstim group). Subjects who failed to solve the problem continued the study and were informed that they will have another opportunity to play the game after the incubation interval.

At the test session, subjects were confronted with the same previously unsolved problem again and were given another chance to find the solution. Additionally, subjects played another new level (level 3 of scenario D) that they had never played before. The order of levels at the test session was counterbalanced across subjects. Unlike in the practice session, there was no time limit to finding the solution. The main interest in the present study was in the solution of the previously unsolved “old” level, therefore performance on the new level was not further considered. Subjects who were able to solve the previously unsolved level in the test session are referred to as “Solvers.” Subjects who failed to solve the level and gave up trying, are referred to as “Non-solvers.” If participants did not solve within 40 min, they were presented the option to give up. If they wanted to continue, they were allowed to do so. However, most subjects gave up upon being presented with this option or shortly thereafter.

To assess performance during the practice session, the training level performance index (TLPI) was calculated, comparing the individual time to solve (TTS) to the mean solving time of each of the four practice levels (TTS1-TTS4) according to the following formula:

$$TLPI_{x}=\frac{1}{\left(\begin{array}{c} \left(\frac{TTS1_{x}}{Mean\left(TTS1\right)}\right)+\\ \left(\frac{TTS2_{x}}{Mean\left(TTS2\right)}\right)+\left(\frac{TTS3_{x}}{Mean\left(TTS3\right)}\right)+\left(\frac{TTS4_{x}}{Mean\left(TTS4\right)}\right) \end{array}\right)}$$

### Sleep Recording

In all sleep groups, 16 electrodes were applied for polysomnographic recordings, including 9 electrodes for electroencephalography (F3, Fz, F4, C3, Cz, C4, P3, Pz, P4), 2 reference electrodes at the mastoids, 2 electrodes for electrooculography (caudolateral of the left and craniolateral of the right eye), 2 electrodes for electromyography (above the mental foramen), and 1 ground electrode in the center of the forehead. Scoring of sleep stages was performed offline for all sleep groups and additionally online for the SWSstim and REMstim groups according to standard criteria (Rechtschaffen and Kales, 1968) as stage 1 sleep, stage 2 sleep, slow wave sleep (SWS, the sum of stage 3 sleep and stage 4 sleep), rapid eye movement (REM) sleep, and time awake. Offline scoring was done blind to the stimulation condition by two independent raters.

### Control Tasks

In order to control for general alertness and cognitive abilities, all participants performed three control tasks twice, once before the practice session and a second time before the test session. To assess participants' vigilance, the vigilance task was applied (Diekelmann et al., 2013). This task required subjects to respond as fast and as accurately as possible to a red dot appearing on the left or right side of the computer screen by pressing the corresponding left or right button. The time interval between appearances of the red dot was 2, 4, 6, 8, or 10 s in random order. The task took about 5 min. Mean reaction times were taken as performance measure. Overall 16 datasets were missing for the vigilance task due to technical issues. The number of valid datasets was *n* = 9 for the Wake group, *n* = 19 for the Sleep group, *n* = 20 for the Wakestim group, *n* = 17 for the SWSstim group, and *n* = 19 for the REMstim group.

To assess working memory capacity, the Digit Span Task (Mueller, 2012) was used, which required subjects to memorize non-repeating sequences of 3–10 digits (0–9). The highest number of digits that subjects were able to recall correctly was taken as performance measure (max. 10). Overall 17 datasets were missing for the working memory task due to technical issues. The number of valid datasets was *n* = 14 for the Wake group, *n* = 16 for the Sleep group, *n* = 21 for the Wakestim group, *n* = 14 for the SWSstim group, and *n* = 18 for the REMstim group.

Subjective sleepiness was assessed with the Stanford sleepiness scale (Hoddes et al., 1973). Subjects were asked to rate their current sleepiness on a scale from 1 (“I feel activated, vitalized, attentive and wide awake”) to 8 (“Sleeping”). Finally, subjective experience on the problem-solving task was assessed with a game experience questionnaire (based on a questionnaire developed by IJsselsteijn et al., 2013). This questionnaire contained 50 items combined into 11 categories assessing the feeling of “competence,” “sensory and imaginative immersion,” “flow,” “tension/annoyance.” “challenge,” “negative affect,” “positive affect,” “positive experience,” “negative experience,” “tiredness” and “returning to reality.” Each item was rated on a scale from 0 (not at all) to 4 (very much).

### Statistical Analysis

The number of subjects who solved the previously unsolved video game problem in the test session was analyzed with χ^2^-Tests after Pearson. Solving speed and the training level performance index (TLPI) were analyzed with independent *t*-Tests in Experiment 1 and one-way ANOVAs in Experiment 2. The control tasks were examined with two-way ANOVAs with the between-subjects factor “group” and the repeated-measures factor “session” (practice/test). Further exploratory three-way ANOVAs included the additional factor “Solvers/Non-Solvers” (reported in Supplementary Materials). Sleep stages were analyzed with independent *t*-Tests in Experiment 1 and one-way ANOVAs in Experiment 2. In case of significant ANOVA effects, *post-hoc* pair-wise *t*-Tests were applied. The level of significance was set to *p* ≤ 0.05.

## Results

### Experiment 1—Sleep and Problem Solving

**Figure 2:**
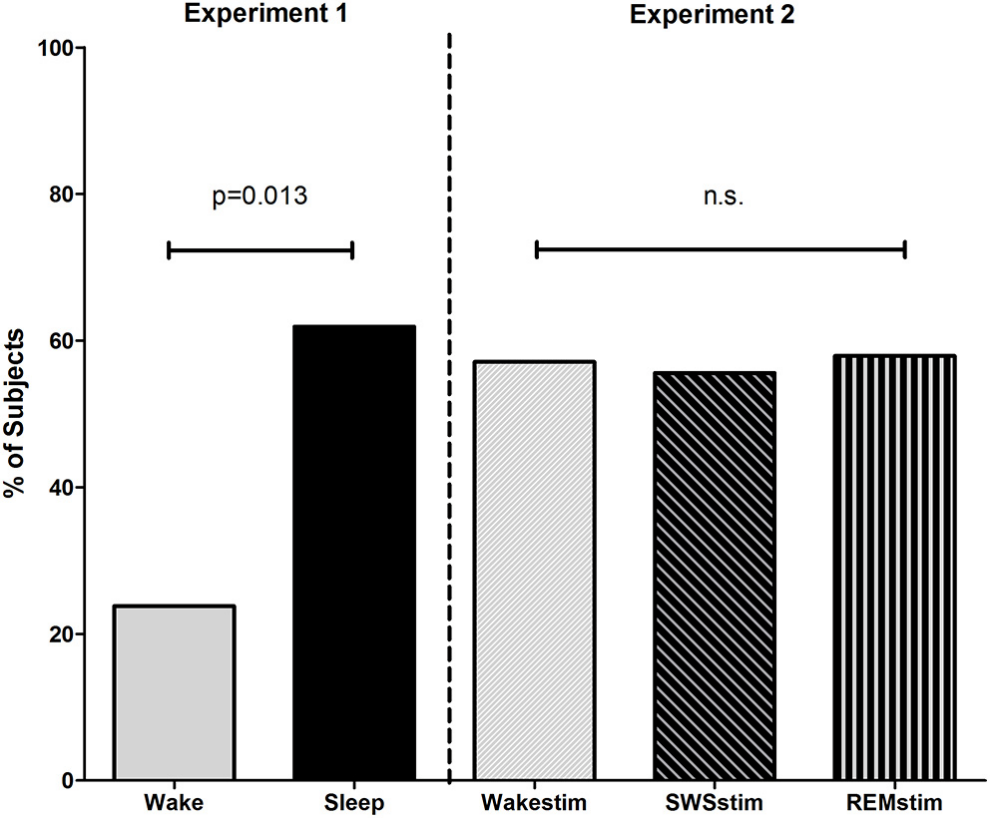
Sleep facilitates problem solving. **Experiment 1**, percentage of subjects who solved the problem after the sleep and wake incubation interval. **Experiment 2**, percentage of subjects who solved the problem after the incubation interval with targeted memory reactivation during wakefulness (Wakestim), SWS (SWSstim) and REM sleep (REMstim).

After being challenged with the previously unsolved video game problem, 61.9% of participants from the Sleep group were able to solve the problem, while only 23.8% of participants from the Wake group solved it (χ^2^ = 6.22; *p* = 0.013, with a medium effect size of Phi = 0.39; Figure 2, Table 2). To evaluate the performance quantitatively, we considered the time needed to solve the problem (solving speed) as a proxy for how well subjects played. However, no difference was found between groups. Subjects from the Sleep group needed 1090.8 ± 82.9 s to solve the problem while the subjects from the Wake group solved the task in 703.0 ± 249.0 s [*t*_(16)_ = 1.16; *p* = 0.26; Table 2]. Although subjects from the Sleep group needed longer to solve the task on a descriptive level, the difference was not significant and the large variance in solving speed makes this measure difficult to interpret. Importantly, subjects in the Sleep group (0.28 ± 0.11) and in the Wake group (0.31 ± 0.10) exhibited comparable training level performance as indicated by the training level performance index [TLPI; *t*_(40)_ = 1.09, *p* = 0.28].

**Table 2:** Problem solving performance and control tasks in Experiment 1 and Experiment 2.

		**Experiment 1**		**Experiment 2**		
	**Session**	**Sleep**	**Wake**	**SWSstim**	**REMstim**	**WakeStim**
# Solvers/Non-solvers		13/8	5/16	8/10	8/11	9/12
Solving speed		1090.8 ± 182.9	703.0 ± 249.0	979.8 ± 227.7	978.4 ± 227.7	1143.3 ± 214.7
TLPI		0.28 ± 0.11	0.31 ± 0.10	0.30 ± 0.10	0.29 ± 0.12	0.28 ± 0.11
Vigilance	Practice	453.8 ± 49.4	457.2 ± 41.6	445.5 ± 38.8	447.7 ± 39.8	442.1 ± 30.6
	Test	456.4 ± 46.7	457.0 ± 42.7	455.8 ± 32.5	443.2 ± 37.8	428.4 ± 20.4
Working memory	Practice	7.5 ± 1.2	7.3 ± 1.7	7.2 ± 1.1	7.5 ± 1.6	6.7 ± 1.3
	Test	7.7 ± 1.3	8.0 ± 1.6	7.8 ± 1.4	7.6 ± 1.5	7.0 ± 1.2
Sleepiness	Practice	3.3 ± 0.9	2.3 ± 0.8	2.9 ± 0.9	3.0 ± 0.7	2.5 ± 0.6
	Test	2.5 ± 0.7	2.9 ± 1.0	2.3 ± 0.8	2.2 ± 0.6	2.9 ± 0.8

*Values are presented as means ± standard deviation. # Solvers/Non-solvers represents the number of subjects who solved the task and those who did not solve the task, respectively. Solving speed represents the time in seconds to solve the task (indicated for solvers only). TLPI represents the training level performance index indicating performance during the four practice levels. Vigilance was measured by the vigilance task and the values presented are the means of reaction times in milliseconds. Working memory was evaluated by the digit span task. Sleepiness was assessed with the Stanford Sleepiness Scale (SSS)*.

Table 2 summarizes the results for the control tasks assessing vigilance, working memory, and subjective sleepiness. There was no difference in vigilance between groups or sessions [main effect group: *F*_(1, 25)_ = 0.11, *p* =0.92; main effect session: *F*(_1, 25)_ = 0.04, *p* = 0.85; interaction group × session: *F*_(1, 40)_ = 0.52, *p* = 0.82]. With regard to working memory, subjects performed generally better at the test session compared to the practice session [main effect session: *F*(_1, 28)_= 5.74, *p* = 0.023] but there was no difference between groups [main effect group: *F*_(1, 28)_= 0.03, *p* = 0.86; interaction group × session: *F*_(1, 28)_= 1.33, *p* = 0.26]. Subjective sleepiness did not differ between groups and sessions [main effect group: *F*_(1, 40)_ = 1.86, *p* = 0.18; main effect session: *F*_(1, 40)_ = 0.30, *p* = 0.40], but the Sleep group showed a stronger reduction of sleepiness from the practice session to the test session compared to the Wake group [interaction group × session: *F*_(1, 40)_ = 23.30, *p* < 0.001]. Importantly, the Sleep and Wake groups were comparable in subjective sleepiness at the test session [*t*_(40)_= 1.38, *p* = 0.17]. Further analyses confirmed that none of the control tasks showed any significant differences between Solvers and Non-Solvers (see Supplementary Table 1).

**Figure 3:**
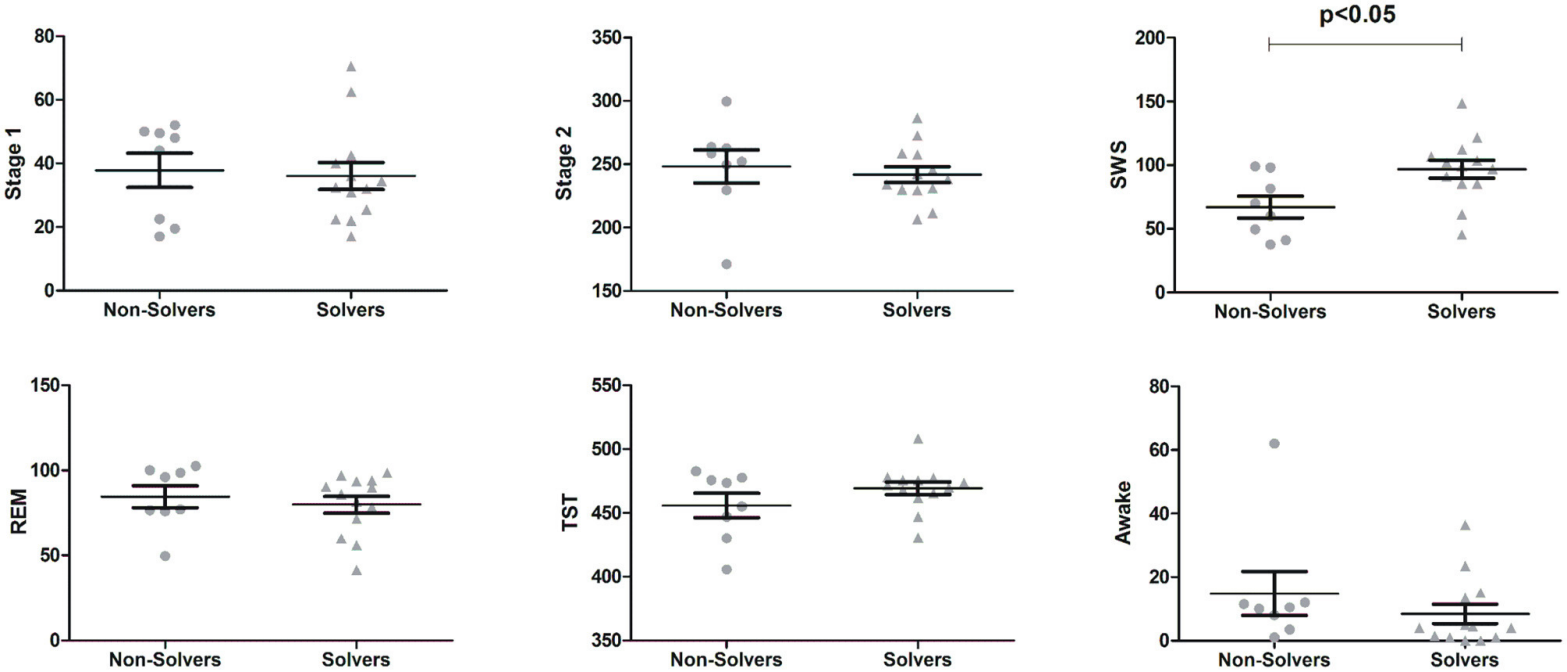
Sleep duration in different sleep stages for subjects from the Sleep group separated by those who solved the problem (Solvers, *n* = 13) and those who did not solve the problem (Non-Solvers, *n* = 8). Means ± Standard Errors of the Means are presented for Stage 1 sleep, Stage 2 sleep, slow wave sleep (SWS), rapid-eye movement (REM) sleep, Total Sleep Time (TST), and time awake after sleep onset.

Subjects from the Sleep group presented overall normal sleep patterns (Figure 3). They slept on average 464.20 ± 4.85 min with 36.71 ± 3.27 min in stage 1, 244.30 ± 6.14 min in stage 2, 85.52 ± 6.24 min in SWS, 81.64 ± 3.84 in REM sleep and 10.9 ± 14.76 awake after sleep onset. To test whether specific sleep stages could explain the performance in problem solving, the duration of single sleep stages was compared between those who solved the problem (Solvers) and those who did not (Non-Solvers). Solvers (*n* = 13) presented higher amounts of SWS in comparison to Non-Solvers [*t*_(19)_= 2.64, *p* = 0.016; Figure 3], with a large effect size (Solvers: 96.88 ± 25.59 min, Non-Solvers: 67.06 ± 36.84 min; Cohen's *d* = 1.19). There were no differences in any of the other sleep stages (all *p* > 0.10; Figure 3).

### Experiment 2—Targeted Memory Reactivation During Sleep for Problem Solving

Problem solving performance did not differ between the groups receiving targeted memory reactivation (TMR) during SWS (SWSstim), REM sleep (REMstim), or wakefulness (Wakestim; χ^2^= 0.02; *p* = 0.99). After the incubation interval with TMR, 55.6% of the participants from the SWSstim group, 57.9% of the REMstim group, and 57.1% of Wakestim group solved the video game problem. The time needed to solve the problem (solving speed) was also comparable between groups [SWSstim: 979.8 ± 227.7 s, REMstim: 978.4 ± 227.7 s, Wakestim: 1143.3 ± 214.7; *F*_(2, 22)_ = 0.19, *p* = 0.83]. Likewise, there were no differences between groups in training level performance [TLPI; SWSstim: 0.30 ± 0.10, REMstim: 0.29 ± 0.12, Wakestim 0.28 ± 0.11; *F*_(2, 55)_= 0.17; *p* = 0.85].

Table 2 summarizes the results for the control tasks assessing vigilance, working memory, and subjective sleepiness. Comparing the three groups in vigilance performance revealed no differences between groups and sessions [main effect group: *F*_(2, 53)_= 1.27, *p* = 0.29; main effect time: *F*_(1, 53)_= 0.47, *p* = 0.50; interaction: *F*_(2, 53)_= 2.97, *p* = 0.06]. For working memory, there was a main effect session [*F*_(1, 50)_= 4.49, *p* = 0.039], indicating generally better performance at the test session compared to the practice session, but this pattern was similarly expressed in all groups [main effect group: *F*_(2, 50)_= 2.26, *p* = 0.12; interaction: *F*_(2, 50)_= 0.60, *p* = 0.55]. For subjective sleepiness, there was a significant interaction between groups and sessions [*F*_(2, 55)_=11.16, *p* < 0.001], suggesting that the Wakestim group was slightly less sleepy during practice [*F*_(2, 55)_=2.49, *p* = 0.09), but sleepier than the other two groups at the test session (*F*_(2, 55)_= 5.83, *p* = 0.005). None of the control tasks showed any significant differences between Solvers and Non-Solvers (see Supplementary Table 2).

Like in Experiment 1, subjects in the SWSstim group and the REMstim showed overall normal sleep parameters (Table 3). To test for differences in specific sleep stages, the duration of single sleep stages was compared between the SWSstim and REMstim groups and between those who solved the problem (Solvers) and who did not (Non-Solvers). For sleep stage 1, there was an interaction effect for group and Solvers/Non-Solvers [F_(1, 33)_= 6.37, *p* = 0.01], with pairwise *post-hoc* comparisons indicating longer stage 1 sleep duration for Non-Solvers in comparison to Solvers in the SWSstim group (*p* = 0.03). Regarding sleep stage 2, there was a main effect for group [*F*_(1, 33)_= 8.52, *p* = 0.006], indicating higher amounts of stage 2 sleep in the REMstim group than in the SWSstim group, with this difference being equally evident in Solvers and Non-Solvers [main effect Solvers/Non-Solvers: *F*_(1, 33)_ = 1.63, *p* = 0.21; interaction: *F*_(1, 33)_= 1.44, *p* = 0.24]. Importantly, neither group received sound stimulation during stage 1 or stage 2 sleep, but only during SWS (in the SWSstim group) and REMsleep (in the REMstim group), respectively. Groups did not differ in the amount of SWS or REM sleep as well as in wake time and total sleep time (*p* > 0.10 for all remaining main effects and interactions; Table 3).

**Table 3:** Sleep stage parameters for subjects from the REMstim and SWSstim groups separately for Solvers and Non-solvers.

	**REMstim**			**SWSstim**		
	**Non-solvers**	**Solvers**	**Entire group**	**Non-solvers**	**Solvers**	**Entire group**
TST (min)	463.59 ± 11.31	458.36 ± 12.49	461.40 ± 2.70	450.25 ± 29.38	452.25 ± 23.57	451.10 ± 6.20
Stage 1 (min)	26.31 ± 14.44	30.00 ± 10.73	27.87 ± 2.94	31.60 ± 8.63	17.25 ± 6.46	25.27 ± 2.48
Stage 2 (min)	259.13 ± 24.98	259.87 ± 27.46	259.40 ± 5.81	223.50 ± 26.27	245.00 ± 25.83	233.10 ± 6.50
SWS (min)	70.45 ± 29.37	66.25 ± 25.33	68.68 ± 6.21	84.65 ± 19.46	79.56 ± 24.58	82.39 ± 5.03
REM (min)	91.13 ± 23.04	81.87 ± 24.63	87.82 ± 5.42	79.60 ± 18.23	89.18 ± 13.73	83.86 ± 3.93
Wake (min)	13.63 ± 20.05	18.68 ± 19.19	15.76 ± 19.29	28.55 ± 17.23	19.37 ± 12.18	24.47 ± 5.50

*Values are presented as means ± standard deviation of time in minutes. TST represents total sleep time, Stage 1 represents stage 1 sleep, Stage 2 represents stage 2 sleep, SWS represents slow wave sleep (the sum of stage 3 and stage 4 sleep), REM represents rapid eye movement sleep, Wake represents time awake after sleep onset*.

Considering that the reactivation procedure started at the onset of SWS and REM sleep, respectively, we also compared the latency to SWS and REM sleep as an indicator of the timing when the participants started receiving reactivation. The SWSstim group and the REMstim group were comparable in SWS latency (SWSstim 22.08 ± 5.21, REMstim 22.95 ± 5.26 min; *p* = 0.88) as well as in REM sleep latency (SWSstim 113.64 ± 26.78, REMstim 118.11 ± 27.10 min; *p* = 0.80). However, the onset of REM sleep was, as expected, significantly later than the onset of SWS (*p* < 0.001), indicating that the reactivation procedure started on average 96 min later in the REMstim group than in the SWSstim group.

### Exploratory Cross-Experiment Comparisons

Although Experiment 1 and Experiment 2 were conceptualized as independent experiments, we ran cross-experiment comparisons for exploratory purposes. Comparing the number of subjects who solved the problem in all five groups revealed no significant difference (χ^2^= 6.24; *p* = 0.18). On a descriptive level, targeted memory reactivation during SWS and REM sleep slightly decreased performance, but the comparison between the three sleep groups was not significant (Sleep vs. SWSstim vs. REMstim: χ^2^= 1.89; *p* = 0.39). On the other hand, targeted memory reactivation during wakefulness seemed to increase problem solving performance, but the difference between the two wake groups did not reach significance (Wake vs. Wakestim: χ^2^= 1.71; *p* = 0.19). Likewise, solving speed (in those subjects who solved the task) was comparable between all five groups [*F*_(4, 38)_ = 0.45, *p* = 0.78] as well as between the three sleep groups [Sleep vs. SWSstim vs. REMstim: *F*_(2, 26)_ = 0.11, *p* = 0.90] and the two wake groups [Wake vs. WakeStim: *t*_(12)_ = 1.27; *p* = 0.22].

To test whether solvers and non-solvers differed with regard to certain participant characteristics, we compared the game experience questionnaire between solvers and non-solvers across all groups (see Supplementary Table 3). These data revealed strong differences between solvers and non-solvers after performance on the problem solving task in the test session, with solvers showing a higher feeling of competence (*p* < 0.001), higher sensory and imaginative immersion (*p* = 0.012), lower feeling of tension/annoyance (*p* < 0.001), lower feeling of challenge (*p* = 0.008), lower negative affect (*p* < 0.001), higher positive affect (*p* < 0.001), higher positive experience (*p* < 0.001), lower negative experience (*p* < 0.001) and lower tiredness (*p* < 0.004) (main effects solvers/non-solvers). These differences were equally pronounced in all experimental groups (all *p* > 0.10 for main effects group and interactions solvers/non-solvers × group). After performance on the problem-solving task in the practice session, however, solvers and non-solvers (i.e., those who would later solve or not solve the task) did not differ in any of the ratings (all *p* > 0.05), speaking against general differences of participant characteristics between solvers and non-solvers.

Comparing the sleep stage distribution among the three sleep groups revealed similar amounts of total sleep time [*F*_(2, 55)_ = 2.04, *p* = 0.14], as well as comparable amounts of SWS [*F*_(2, 55)_ = 2.30, *p* = 0.11] and REM sleep [*F*_(2, 55)_ = 0.51, *p* = 0.61], suggesting that the auditory TMR procedure applied during SWS and REM sleep, respectively, did not affect the amount of sleep spent in those sleep stages. However, there were significant differences between groups in wake time [*F*_(2, 55)_ = 3.32, *p* = 0.044], stage 1 sleep [*F*_(2, 55)_ = 4.21, *p* = 0.02], and stage 2 sleep [*F*_(2, 55)_ = 4.44, *p* = 0.016]. *Post-hoc* comparisons revealed higher amounts of wake time (*p* = 0.008) and lower amounts of stage 1 (*p* = 0.01) in the SWSstim group compared to the Sleep group, as well as lower amounts of stage 2 in the SWSstim group compared to the REMstim group (*p* = 0.005; for all other comparisons: *p* > 0.05). Moreover, comparing the amounts of SWS for Solvers of the Sleep group with all other participants from the SWSstim and REMstim groups, revealed significantly more SWS for the Sleep group Solvers (*p* = 0.01).

Additionally, we performed analyses on movement time (extended arousals of more than half an epoch, in minutes) and the overall number of sleep epochs with arousals as potential indicators of sleep disturbances due to the reactivation procedure. With regard to movement time, the three sleep groups differed significantly [Sleep 5.17 ± 0.78, SWSstim 2.08 ± 0.32, REMstim 1.84 ± 0.23 min; *F*(2, 55) = 12.42, *p* < 0.001]. However, *post-hoc* tests revealed that both reactivation groups showed less movement time than the sleep group without reactivation (SWSstim vs. Sleep, *p* = 0.001; REMstim vs. Sleep, *p* < 0.001), whereas the reactivation groups did not differ (SWSstim vs. REMstim, *p* = 0.55). The number of epochs with arousals likewise differed significantly between groups [Sleep 68.10 ± 4.26, SWSstim 46.33 ± 3.87, REMstim 62.15 ± 3.69; *F*(2, 55) = 7.83, *p* = 0.001], with the SWSstim group showing fewer arousals than the sleep group without reactivation (*p* < 0.001) and also fewer arousals than the REMstim group (*p* = 0.005), whereas there was no difference between the REMstim group and the sleep group without reactivation (*p* = 0.30).

## Discussion

In this study, we found that a whole night of sleep facilitates generating a solution to a previously unsolved problem in the B-SEM video game task. This benefit of sleep was particularly associated with SWS, with those subjects who solved the problem showing higher amounts of SWS during the incubation interval. However, problem solving did not additionally benefit from targeted memory reactivation (TMR) during the incubation interval, neither during SWS nor REM sleep.

Our Experiment 1, showing a benefit of sleep for problem solving, replicates and extends previous evidence from Beijamini et al. (2014). In that study, participants had a higher chance to solve B-SEM problem following an afternoon nap of ~67 min compared to subjects who stayed awake during this time. The subjects who obtained SWS during the nap were also more likely to find the solution than subjects without any SWS. However, subjects obtained only ~10 minutes of SWS during the nap, which is why a comparison between the absolute amount of SWS between solvers and non-solvers was not possible in that study. Here, we extend the findings by Beijamini et al. (2014) in showing that (1) not only a nap but also a full night of sleep increases problem solving, and (2) that subjects with higher amounts of SWS had a higher chance of solving the problem. Together, these findings suggest that SWS-associated processes are mechanistically involved in the generation of insight into problem-solving tasks. This assumption is in keeping with previous evidence, showing that insight in the Number Reduction Task (NRT), a task that requires subjects to gain insight into hidden regularities, is higher after early SWS-rich sleep compared to late REM sleep-rich sleep (Yordanova et al., 2008). In those studies, slow spindle/alpha activity particularly during SWS predicted the transition from implicit knowledge to explicit insight (Yordanova et al., 2012). Yet, there are also conflicting results, e.g., observations that problem solving in the Remote Associates Test (RAT), requiring subjects to identify semantic relations between words, is linked to REM sleep rather than SWS (Cai et al., 2009). Two other studies, comparing a wide range of different tasks, including magic tricks, classical insight-type of tasks, change detection, anagrams etc. entirely failed to reveal any beneficial effects of a post-training nap on solving the problems (Brodt et al., 2018; Schonauer et al., 2018). With the exception of solving riddles, task performance in those experiments did not even benefit from an incubation period itself (Brodt et al., 2018). The divergent outcomes between those and the present study are difficult to explain. There may have been differences in the subject samples, e.g., with regard to gender distribution. About half of the participants in Brodt et al. (2018) and Schonauer et al. (2018) were male, whereas > 80% of participants in the present Experiment 1 were female, and it is well known that there are gender differences in cognitive functions, including problem solving (Johnson, 1984; Hyde, 2014). Also, while subjects were presented with a large number of different problems with only limited time to solve each problem in Brodt et al. (2018) and Schonauer et al. (2018), the present study employed only one problem-solving task, i.e., the B-SEM task, for which subjects spent a considerable amount of time trying to find a solution. According to participants' anecdotal reports, the B-SEM task is quite engaging and at times also frustrating, which might have increased the relevance and (emotional) salience of the task, factors that are known to boost sleep-dependent processing (Fischer and Born, 2009; Wilhelm et al., 2011; Payne et al., 2012; Bennion et al., 2016). Moreover, the role of sleep may strongly depend on the type of problem-solving task and the underlying processes mediating insight into the solution (Lewis et al., 2018; Lerner and Gluck, 2019; Lutz and Born, 2019). Lewis and colleagues (Lewis et al., 2018) proposed that NREM sleep, including SWS, facilitates the abstraction of common rules and regularities from learned information, whereas REM sleep supports the generation of novel associations. Accordingly, depending on the process primarily involved in solving a specific problem, finding the solution may either benefit from SWS or from REM sleep or from both or may not benefit from sleep at all if other so far unknown processes are involved. In fact, the B-SEM task is one of the few typical complex insight problem solving tasks for which a benefit of sleep has been consistently shown^1^. To examine this question in more detail, it will be essential to advance our understanding of the specific processes underlying problem solving in different tasks (Sio and Ormerod, 2009; Gilhooly, 2016).

One limitation of the previous study by Beijamini et al. (2014) was the heterogeneity of the participants' performance levels at training. Subjects were allowed to play freely until they were unable to solve one level and this level then served as the problem-solving task the subjects attempted to solve again after the nap or no nap interval. Thus, the difficulty of the problem level at testing varied considerably, preventing a straight forward comparison of performance in the nap and no nap groups. In the present study, we mitigated this limitation by presenting all subjects with the same video game levels during practice as well as at testing, achieving comparable pre-sleep and post-sleep difficulty of the unsolved challenge. Consequently, here we could also determine solving speed as a more complex quantitative measure of problem solving, in addition to the simple measure of the number of subjects solving the task. However, this measure did not yield any differences between sleep and wake groups, neither between those who solved the problem and those who did not. This finding suggests that the process of problem solving in the video game task is more likely to rely on “all-or-none” sudden insight into the solution that can occur at any point of the task, rather than a more incremental analytical solution generation process (Smith and Kounios, 1996; Kounios and Beeman, 2014; Laukkonen and Tangen, 2018). Alternatively, other processes such as motivation, sleepiness, logical reasoning capacities or circadian variations may have additionally affected the time needed to solve the problem, thereby masking the effect of solution generation processes on solving speed. Circadian differences may have emerged due to the testing session taking place at different times of day for the sleep group (testing in the morning) and the wake group (testing in the evening). Differences in general participant characteristics like motivation, interest in the task, or perseverance, may have further influenced the likelihood of solving the task or giving up trying. Although we found that non-solvers reported overall more negative feelings after the video game task in the test session, they did not differ in any aspect of video game experience during the practice session, suggesting that negative feelings in the test session were due to not finding the solution rather than general differences in subject characteristics. Moreover, we did not observe any differences between groups in video game experience, general alertness, vigilance and working memory capacity. Nevertheless, future studies should consider these factors and examine the underlying mechanisms of solution generation more systematically.

Contrary to our hypothesis, we did not observe any beneficial effects of targeted memory reactivation (TMR) on problem solving. Although there is convincing evidence that TMR facilitates memory consolidation in a wide range of experimental tasks (Klinzing and Diekelmann, 2019; Hu et al., 2020), the effects of TMR on problem solving are not well understood. In one study, Ritter and colleagues paired a specific odor with a problem based on the Unusual Uses Task (Ritter et al., 2012). During sleep in the following night, subjects were presented either with the same odor again, a different odor, or no odor at all. In the next morning, participants who had received the same odor during sleep generated more creative solutions to the problem and were better at selecting the most creative solution. In another study, Sanders and colleagues presented subjects with different puzzles, each associated with an arbitrary sound (Sanders et al., 2019). Half of the sounds of the unsolved puzzles were then presented again during subsequent sleep, specifically during SWS. In the next morning, participants solved more of those puzzles for which the associated sounds were played (cued) during sleep compared to the uncued puzzles. These findings suggest that TMR can increase problem solving after sleep in some conditions. We can only speculate why we did not observe any beneficial effects of TMR on problem solving in the present study. On a descriptive level, the exploratory cross-experiment comparisons even pointed toward slightly decreased problem solving performance after TMR during sleep compared to the undisturbed sleep group. Although this difference did not reach significance, it could be speculated that in our experimental design, the TMR procedure biased processing during sleep toward a strengthening of memories from the video game along with the successful strategies applied during the practice session. In order to find the solution for the problem, however, it was necessary to relax task constraints and do something entirely different than the strategies that were previously successful (Knoblich et al., 1999; Ormerod et al., 2002). By boosting the strengthening of the learned strategies, TMR during sleep might have hindered associative processing and the creative recombination of encoded elements that could lead to new associations and insight into the solution. In support of this idea, Landmann et al. (2016) showed better retention of previously solved problems after sleep but no effect of sleep on finding the solution for previously unsolved problems in the Remote Associates Test. Although that study did not include TMR, it is conceivable that TMR biases processing toward sleep's strengthening function rather than the generation of novel associations. The TMR protocol of the present study might have contributed to such a bias, considering that the TMR stimuli were composed of inherent sounds from the video game, which might have particularly reactivated successful strategies from the practice session, possibly hindering creative new solutions.

Alternatively, the auditory TMR protocol of the present study might have affected sleep quality and disturbed ongoing consolidation processes, particularly during SWS, thereby counteracting the beneficial effects on problem solving. The exploratory cross-experiment comparisons between undisturbed sleep and the two TMR conditions revealed that the SWSstim group showed higher amounts of wake time and lower amounts of stage 1 sleep than the undisturbed sleep group. Additional arousal analyses, on the other hand, revealed less movement time in both TMR conditions as well as fewer epochs with arousals in the SWSstim group compared to the undisturbed sleep group. Moreover, total sleep time as well as the amount of SWS and REM sleep was comparable between undisturbed sleep and the TMR groups. Collectively, the present data do not provide strong evidence for sleep disturbances due to the TMR protocol, but we cannot exclude more subtle disturbances at the level of more fine-grained EEG analyses. Another limitation of the present study is the timing of TMR in the SWSstim and REMstim groups. Because REM sleep naturally occurs later in the night, the TMR groups differed with regard to the onset of the reactivation protocol, with the REMstim group receiving the first TMR cues about 90 min later than the SWSstim group. However, since we did not observe any behavioral effects of TMR, we consider it unlikely that this difference in timing affected our results. Indeed, our observation of higher amounts of SWS in Solvers of the undisturbed sleep group compared to both TMR groups, suggests that the most essential factor for a beneficial effect of sleep for problem solving may be high amounts of undisturbed SWS. Future studies on TMR and problem solving should more systematically control for sleep disturbances and examine more fine-grained measures of sleep quality and sleep-related information processing, such as spindles and slow wave activity.

On a descriptive level, the exploratory cross-experiment comparisons further pointed toward increased problem solving after TMR during wakefulness. Although wake reactivation was not the main focus of the present study, it can be speculated that this increase in performance is a result of memory strengthening upon reconsolidation processes. Previous evidence from the reconsolidation literature suggests that reactivation during wakefulness labilizes memory traces, and these memory traces can then become either weakened or strengthened depending on the number of reactivation cues and whether or not interference is presented after reactivation. Forcato and colleagues showed that labilized memories are weakened if only one reactivation cue is presented along with interference learning after the cue, whereas the memories are strengthened if more than one reactivation cue is presented without any interference learning (Forcato et al., 2011, 2014). The paradigm of the present study resembles the latter case, considering that the Wakestim group received numerous reactivation cues and no interference. Thus, TMR during wakefulness might strengthen memories in a reconsolidation-like process, which should be subject to further investigation.

## Data Availability Statement

The raw data supporting the conclusions of this article will be made available by the authors upon request, without undue reservation.

## Ethics Statement

The studies involving human participants were reviewed and approved by the Ethics committee of the medical faculty of the University of Tübingen. The participants provided their written informed consent to participate in this study.

## Author Contributions

FB conceived, designed and planned the study, collected and analyzed the data, and wrote the manuscript. AV collected and analyzed the data. RJ collected and analyzed the data. JB designed the study and wrote the manuscript. SD designed and planned the study, analyzed the data and wrote the manuscript. All authors discussed the data and revised the manuscript.

## Conflict of Interest

The authors declare that the research was conducted in the absence of any commercial or financial relationships that could be construed as a potential conflict of interest.

## Footnotes

^1^Note that Beijamini and colleagues replicated the beneficial effect of a nap for problem solving in the video game task once again (unpublished, in preparation).
