# Supplementary material for: Sleep Facilitates Problem Solving With No Additional Gain Through Targeted Memory Reactivation
Source: Front Behav Neurosci. 2021 Mar 3;15:645110. doi: 10.3389/fnbeh.2021.645110 (PMC7965947; doi:10.3389/fnbeh.2021.645110)
Supplement: Supplementary file 1 [file Data_Sheet_2.PDF]

Supplementary Material

1 Supplementary analyses

To test whether there were differences in control tasks between Solvers and Non-solvers in the Sleep and Wake group of Experiment 1, three-way ANOVAs were performed with the factors group (Sleep/Wake), session (practice/testing) and Solvers/Non-Solvers. For vigilance and working memory performance, there were no significant differences ($p>0.09$ for all main effects and interactions; Supplementary Table 1). For subjective sleepiness, there was an interaction for group × session ($p<0.001$), with the Sleep group showing a stronger reduction of sleepiness from the practice session to the test session, but this effect was comparable for Solvers and Non-Solvers ($p\geq0.25$ for all remaining main effects and interactions). This analysis confirms that the observed difference in Solvers and Non-Solvers between groups was not related to the control factors vigilance, working memory capacity or subjective sleepiness.

We also tested for differences in control tasks between Solvers and Non-solvers in Experiment 2, running three-way ANOVAs with the factors group (SWSstim/REMstim/Wakestim), session (practice/testing) and Solvers/Non-Solvers. For vigilance and working memory performance, there were no significant differences ($p>0.05$ for all main effects and interactions; Supplementary Table 2). For subjective sleepiness, there was a main effect of session ($p=0.009$) and an interaction for group × session ($p<0.001$), with the sleep groups (SWSstim and REMstim) showing a stronger reduction of sleepiness from the practice session to the test session than the Wakestim group, but this effect was comparable for Solvers and Non-Solvers ($p>0.39$ for all remaining main effects and interactions).

To test whether Solvers and Non-solvers differed with regard to certain participant characteristics, we compared the game experience questionnaire between Solvers and Non-solvers across all groups, running two-way ANOVAs with the factors group (Sleep/Wake/SWSstim/REMstim/Wakestim) and Solvers/Non-Solvers, separately for the practice and test session (Supplementary Table 3). These data revealed strong differences between Solvers and Non-solvers after performance on the problem solving task in the test session, with Solvers showing a higher feeling of competence ($p < 0.001$), higher sensory and imaginative immersion ($p = 0.012$), lower feeling of tension/annoyance ($p < 0.001$), lower feeling of challenge ($p = 0.008$), lower negative affect ($p < 0.001$), higher positive affect ($p < 0.001$), higher positive experience ($p < 0.001$), lower negative experience ($p < 0.001$) and lower tiredness ($p < 0.004$) (main effects Solvers/Non-solvers). These differences were equally pronounced in all experimental groups (all $p > 0.10$ for main effects group and interactions Solvers/Non-solvers x group). After performance on the problem solving task in the practice session, however, Solvers and Non-solvers (i.e. those who would later solve or not solve the task) did not differ in any of the ratings ($p > 0.05$ for all main effects and interactions).

2 Supplementary Tables

Supplementary Table 1. Control task comparisons between groups and subgroups (Non-solvers and Solvers) in Experiment 1

		Wake			Sleep		
	**Session**	Non-Solvers	Solvers	Entire group	Non-Solvers	Solvers	Entire group
**N**		16	5	21	8	13	21
**Vigilance**	**Practice**	459.2±44.6	443.4+	457.2± 41.6	454.1±62.5	453.6±40.6	453.8±49.4
	**Test**	456.0±46.0	464.0+	457.0± 42.7	461.9±64.9	452.5±30.3	456.4±46.7
**Working Memory**	**Practice**	7.3±1.7	7.7±2.1	7.3±1.7	7.7±1.1	7.3±1.3	7.5±1.2
	**Test**	8.1±1.6	8.0±1.4	8.0±1.6	8.0±1.1	7.6±1.6	7.7±1.3
**Sleepiness**	**Practice**	2.3±0.8	2.2±0.8	2.3±0.8	3.1±0.6	3.4±1.0	3.3±0.9
	**Test**	2.9±1.1	2.8±0.8	2.8±1.0	2.3±0.9	2.6±0.7	2.5±0.7

Values are presented as Means ± Standard deviation. Vigilance was measured by the vigilance task and the values presented are the means of reaction times in milliseconds. Working memory was evaluated by the Digit Span Task. Sleepiness was assessed with the Stanford Sleepiness Scale (SSS).
+ Note that there was only one dataset for the vigilance task in the subgroup of Solvers

Supplementary Table 2. Control task comparisons between groups and subgroups (Non-solvers and Solvers) in Experiment 2

		Wakestim			REMstim			SWSstim		
	**Session**	Non-Solvers	Solvers	Entire group	Non-Solvers	Solvers	Entire group	Non-Solvers	Solvers	Entire group
**N**		12	9	21	11	8	19	10	8	18
**Vigilance**	**Practice**	442.5±33.6	441.5±28.4	442.1±30.6	462.8±46.1	426.8±13.7	447.7±39.8	441.1±38.8	452.5±40.9	445.5±38.8
	**Test**	427.6±20.9	429.3±20.9	428.4±20.4	454.1±36.9	428.0±35.6	443.2±37.8	448.2±28.1	466.8±37.4	455.8±32.5
**Working Memory**	**Practice**	6.8±1.5	6.6±1.1	6.7±1.3	7.6±1.8	7.5±1.4	7.6±1.6	7.6±1.0	7.0±1.2	7.3±1.1
	**Test**	7.3±1.4	6.7±0.7	7.0±1.2	7.8±1.8	7.5±1.2	7.7±1.5	8.3±1.5	7.4±1.3	7.9±1.4
**Sleepiness**	**Practice**	2.6±0.7	2.4±0.5	2.5±0.6	3.1±0.9	2.9±0.4	3.0±0.7	2.9±0.9	3.0±0.9	2.9±0.9
	**Test**	3.2±0.7	2.7±0.9	2.9±0.8	2.2±0.6	2.1±0.6	2.2±0.6	2.4±0.8	2.4±0.9	2.3±0.8

Values are presented as Means ± Standard deviation. Vigilance was measured by the vigilance task and the values presented are the means of reaction times in milliseconds. Working memory was evaluated by the Digit Span Task. Sleepiness was assessed with the Stanford Sleepiness Scale (SSS)

Supplementary Table 3. Ratings in the game experience questionnaire for all groups and subgroups (Non-solvers and Solvers)

		Wake		Sleep		WakeStim		REMstim		SWSstim	
	**Session**	Non-Solvers	Solvers	Non-Solvers	Solvers	Non-Solvers	Solvers	Non-Solvers	Solvers	Non-Solvers	Solvers
**N**		16	5	8	13	12	9	11	8	10	8
**Comp**	**Practice**	1.48 ± 0.23	1.60 ± 0.41	0.95 ± 0.33	1.19 ± 0.26	0.73 ± 0.27	1.98 ± 0.31	1.33 ± 0.28	1.00 ± 0.34	1.48 ± 0.29	1.50 ± 0.33
	**Test**	1.04 ± 0.23	2.32 ± 0.42	0.23 ± 0.33	1.68 ± 0.26	0.55 ± 0.27	2.29 ± 0.31	1.00 ± 0.28	1.83 ± 0.33	0.76 ± 0.29	2.03 ± 0.33
**Sens**	**Practice**	1.54 ± 0.18	1.37 ± 0.33	1.42 ± 0.26	1.63 ± 0.21	1.29 ± 0.21	1.72 ± 0.25	1.61 ± 0.22	1.56 ± 0.26	1.70 ± 0.23	1.96 ± 0.26
	**Test**	1.26 ± 0.18	1.37 ± 0.33	1.04 ± 0.26	1.63 ± 0.20	1.11 ± 0.21	1.74 ± 0.24	1.42 ± 0.22	1.55 ± 0.26	1.33 ± 0.23	1.90 ± 0.26
**Flow**	**Practice**	1.93 ± 0.19	2.12 ± 0.33	1.78 ± 0.26	2.34 ± 0.21	2.08 ± 0.21	2.24 ± 0.25	2.44 ± 0.22	2.50 ± 0.26	2.04 ± 0.24	2.53 ± 0.26
	**Test**	1.73 ± 0.22	2.08 ± 0.40	1.75 ± 0.32	2.11 ± 0.25	2.15 ± 0.26	2.02 ± 0.30	2.11 ± 0.27	2.03 ± 0.32	1.84 ± 0.28	2.03 ± 0.32
**Tens**	**Practice**	1.04 ± 0.28	0.60 ± 0.49	1.38 ± 0.39	1.74 ± 0.31	1.67 ± 0.32	0.89 ± 0.37	1.27 ± 0.33	1.33 ± 0.39	0.67 ± 0.35	1.42 ± 0.39
	**Test**	2.22 ± 0.30	0.60 ± 0.52	2.79 ± 0.41	1.21 ± 0.32	2.83 ± 0.34	1.00 ± 0.39	2.36 ± 0.35	1.25 ± 0.41	1.60 ± 0.37	0.92 ± 0.41
**Chal**	**Practice**	1.70 ± 0.19	1.16 ± 0.35	1.85 ± 0.27	2.14 ± 0.21	2.03 ± 0.22	1.64 ± 0.26	1.84 ± 0.23	2.13 ± 0.27	1.64 ± 0.24	2.25 ± 0.27
	**Test**	2.00 ± 0.21	1.20 ± 0.37	2.23 ± 0.29	1.88 ± 0.23	2.33 ± 0.24	1.38 ± 0.28	2.02 ± 0.25	2.00 ± 0.29	1.76 ± 0.26	1.53 ± 0.29
**Neg A**	**Practice**	0.95 ± 0.20	0.30 ± 0.36	0.94 ± 0.28	0.90 ± 0.22	1.19 ± 0.23	0.89 ± 0.26	1.09 ± 0.24	1.06 ± 0.28	0.93 ± 0.25	1.03 ± 0.28
	**Test**	1.55 ± 0.20	0.55 ± 0.36	1.91 ± 0.29	0.60 ± 0.23	1.65 ± 0.24	0.89 ± 0.27	1.80 ± 0.25	0.72 ± 0.29	1.50 ± 0.26	0.60 ± 0.29
**Pos A**	**Practice**	2.19 ± 0.24	2.48 ± 0.43	1.73 ± 0.34	2.17 ± 0.26	1.52 ± 0.28	2.69 ± 0.32	2.15 ± 0.29	1.90 ± 0.34	2.26 ± 0.30	2.08 ± 0.34
	**Test**	1.25 ± 0.24	2.68 ± 0.42	0.75 ± 0.33	2.08 ± 0.26	0.93 ± 0.27	3.00 ± 0.32	1.47 ± 0.29	2.40 ± 0.33	1.30 ± 0.32	2.40 ± 0.33

**Pos E**	**Practice**	1.23 ± 0.18	1.43 ± 0.33	0.79 ± 0.26	1.05 ± 0.20	1.10 ± 0.21	1.69 ± 0.24	1.32 ± 0.22	1.10 ± 0.26	1.40 ± 0.23	1.25 ± 0.26
	**Test**	0.81 ± 0.23	2.17 ± 0.41	0.46 ± 0.32	2.15 ± 0.25	0.61 ± 0.26	2.30 ± 0.30	1.47 ± 0.27	2.13 ± 0.32	0.87 ± 0.29	2.17 ± 0.34
**Neg E**	**Practice**	0.74 ± 0.18	0.63 ± 0.32	0.81 ± 0.26	0.69 ± 0.20	1.01 ± 0.21	0.50 ± 0.24	0.82 ± 0.22	0.92 ± 0.26	0.67 ± 0.23	0.79 ± 0.26
	**Test**	1.21 ± 0.19	0.67 ± 0.34	1.21 ± 0.27	0.35 ± 0.21	0.92 ± 0.24	0.62 ± 0.29	1.24 ± 0.23	0.65 ± 0.27	1.39 ± 0.22	0.44 ± 0.26
**Tired**	**Practice**	0.81 ± 0.22	0.60 ± 0.40	1.06 ± 0.32	1.35 ± 0.25	0.79 ± 0.26	0.50 ± 0.30	1.32 ± 0.27	0.94 ± 0.32	1.15 ± 0.28	1.56 ± 0.32
	**Test**	1.63 ± 0.25	1.30 ± 0.45	1.25 ± 0.35	0.77 ± 0.28	1.79 ± 0.29	0.56 ± 0.33	1.09 ± 0.30	0.13 ± 0.35	1.10 ± 0.31	1.00 ± 0.38
**Real**	**Practice**	0.35 ± 0.15	0.60 ± 0.26	0.58 ± 0.21	0.74 ± 0.16	0.44 ± 0.17	0.22 ± 0.19	0.36 ± 0.18	0.38 ± 0.21	0.57 ± 0.18	0.67 ± 0.21
	**Test**	0.44 ± 0.13	0.33 ± 0.24	0.42 ± 0.19	0.28 ± 0.15	0.36 ± 0.15	0.44 ± 0.18	0.46 ± 0.16	0.13 ± 0.19	0.37 ± 0.17	0.29 ± 0.20

Values are presented as Means ± Standard error of the means. Comp, competence; Sens, sensory and imaginative immersion; Flow, flow; Tens, tension/annoyance; Chal, challenge; Neg A, negative affect; Pos A, positive affect; Pos E, positive experience; Neg E, negative experience; Tired, tiredness; Real, returning to reality.
